# MCC/Eisosomes Regulate Cell Wall Synthesis and Stress Responses in Fungi

**DOI:** 10.3390/jof3040061

**Published:** 2017-11-03

**Authors:** Jenna E. Foderaro, Lois M. Douglas, James B. Konopka

**Affiliations:** Department of Molecular Genetics and Microbiology, Stony Brook University, Stony Brook, NY 11794-5222, USA; jenna.foderaro@stonybrook.edu (J.E.F.); lmardouglas@gmail.com (L.M.D.)

**Keywords:** *Candida albicans*, fungal, cell wall, chitin, β-glucan, morphogenesis, eisosome, Membrane Compartment of Can1 (MCC) domain

## Abstract

The fungal plasma membrane is critical for cell wall synthesis and other important processes including nutrient uptake, secretion, endocytosis, morphogenesis, and response to stress. To coordinate these diverse functions, the plasma membrane is organized into specialized compartments that vary in size, stability, and composition. One recently identified domain known as the Membrane Compartment of Can1 (MCC)/eisosome is distinctive in that it corresponds to a furrow-like invagination in the plasma membrane. MCC/eisosomes have been shown to be formed by the Bin/Amphiphysin/Rvs (BAR) domain proteins Lsp1 and Pil1 in a range of fungi. MCC/eisosome domains influence multiple cellular functions; but a very pronounced defect in cell wall synthesis has been observed for mutants with defects in MCC/eisosomes in some yeast species. For example, *Candida albicans* MCC/eisosome mutants display abnormal spatial regulation of cell wall synthesis, including large invaginations and altered chemical composition of the walls. Recent studies indicate that MCC/eisosomes affect cell wall synthesis in part by regulating the levels of the key regulatory lipid phosphatidylinositol 4,5-bisphosphate (PI_4,5_P_2_) in the plasma membrane. One general way MCC/eisosomes function is by acting as protected islands in the plasma membrane, since these domains are very stable. They also act as scaffolds to recruit >20 proteins. Genetic studies aimed at defining the function of the MCC/eisosome proteins have identified important roles in resistance to stress, such as resistance to oxidative stress mediated by the flavodoxin-like proteins Pst1, Pst2, Pst3 and Ycp4. Thus, MCC/eisosomes play multiple roles in plasma membrane organization that protect fungal cells from the environment.

## 1. Introduction

The plasma membrane has a complex mission to form a protective barrier around the cell while also mediating a wide range of dynamic functions. The plasma membrane is organized into distinct compartments to coordinate these diverse activities [1,2,3,4,5,6,7,8]. For example, cell wall material and proteins get secreted at sites that are distinct from where endocytosis occurs. The membrane compartments also vary in size and stability. For example, small domains can be formed by the clustering of lipids, and even larger domains can form if they are stabilized by proteins, such as the formation of septin rings at sites of septation [8]. Many membrane domains are transient, such as sites of secretion or endocytosis that last just a few minutes. However, recently described Membrane Compartment of Can1 (MCC)/eisosome domains are very stable structures that are revealing novel roles for the plasma membrane. Therefore, as part of this special issue on the fungal cell wall, we will review the emerging roles of MCC/eisosomes with an emphasis on their influence on cell wall synthesis and stress responses.

**Figure 1:**
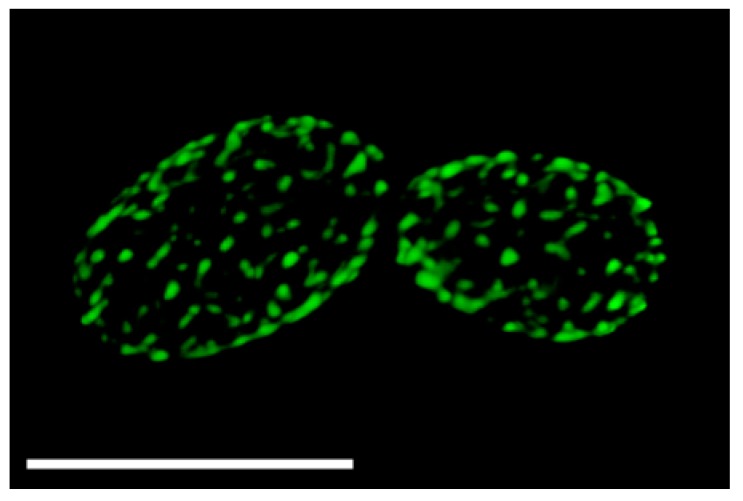
*C. albicans* Membrane Compartment of Can1 (MCC)/eisosome domains visualized with Sur7-GFP (Green Fluorescent Protein). *C. albicans* cells producing a Sur7-GFP fusion protein were analyzed by deconvolution of images taken by fluorescence microscopy. Note the presence of the punctate patches that correspond to the MCC/eisosome domains. Bar, 5 µm.

Studies with *Saccharomyces cerevisiae* revealed that the fungal plasma membrane can be generally divided into two major domains. A large portion of the plasma membrane is referred to as the MCP (Membrane Compartment of Pma1, the plasma membrane H+ ATPase). The MCP is an active zone in which proteins diffuse and dynamic processes occur, such as secretion, endocytosis, and cell wall synthesis [9,10]. In contrast, there are about 50 MCC domains (named for the Can1 arginine permease) per cell that appear by fluorescence microscopy as a series of patches in the plasma membrane [10,11]. An example of the punctate localization of the MCC protein Sur7 is shown in Figure 1. The MCC domains were subsequently shown to be associated with cytoplasmic proteins that form a complex called the eisosome [12]. Since the MCC and eisosome are adjacent structures we will refer to them as MCC/eisosomes. These domains are distinct relative to other plasma membrane compartments in that they correspond to stable furrows in the plasma membrane that are about 50 nm deep and ~300 nm long [13,14]. Interestingly, similar membrane furrows have been detected in a wide range of organisms, including fungi, algae, and lichens, suggesting that MCC/eisosomes are broadly conserved in organisms with a cell wall [15].

Mutational analysis indicates that MCC/eisosome domains are needed for proper spatial regulation of cell wall synthesis and other functions, including responses to stress. However, it does not seem likely that MCC/eisosomes have a direct role in these dynamic processes, since these immobile punctate patches are distinct from sites of endocytosis, secretion, and cell wall synthesis [7,13]. Therefore, we will review recent data on the mechanisms by which MCC/eisosomes promote plasma membrane function, including their ability to act as protected domains that are shielded from endocytosis [10,11,16,17,18], as regulators of lipid homeostasis [19,20,21,22], and possibly as reservoirs for expansion of the plasma membrane [23,24].

## 2. MCC/Eisosome Domain Assembly and Structure

### 2.1. Discovery That MCC Domains, Eisosomes, and Plasma Membrane Furrows Correspond to the Same Membrane Compartment

Three lines of work converged to define the structure of the MCC/eisosome domains. In one approach, localization of the Sur7 protein and the Can1 arginine permease in *S. cerevisiae* revealed that these integral plasma membrane proteins localized to punctate patches [7,25,26,27,28]. Their patchy distribution was spatially distinct from cortical actin patches in the plasma membrane, and also contrasted with transient actin patches in that their localization was very stable. This provided the first evidence that Sur7 and Can1 correspond to a novel type of membrane domain, the MCC [25,28]. Subsequently, additional integral membrane proteins were discovered to localize to the MCC, mainly by screening of Green Fluorescent Protein (GFP)-tagged proteins. This included other nutrient transporters and members of two different families of tetraspan proteins [21,28,29]. A description of the known MCC/eisosome proteins is presented in Table 1.

**Figure 2:**
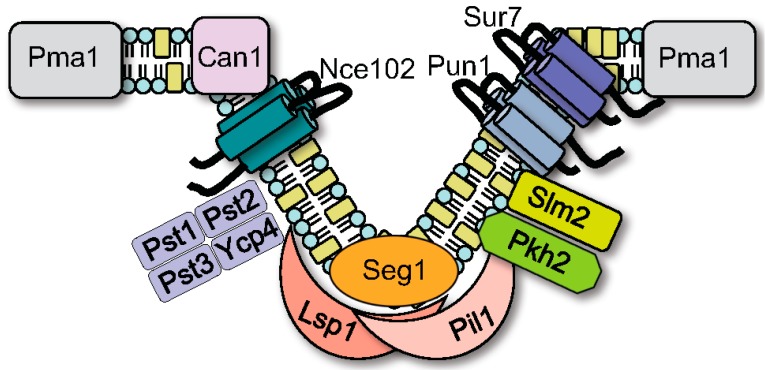
Model for MCC/eisosome structure. Representatives of the approximately 30 proteins that localize to MCC/eisosomes in *S. cerevisiae* are shown.

Other studies examining peripheral membrane proteins discovered that Pil1 and its paralog Lsp1 form a complex on the cytoplasmic side of the MCC [12]. As will be described in more detail below, Pil1 and Lsp1 promote MCC/eisosome formation by binding the plasma membrane through their BAR (Bin/Amphiphysin/Rvs) domains and assembling into long filaments to stabilize these structures [39]. This cluster of proteins was termed the eisosome, a fusion of the Greek “*eis*”, meaning into or portal, and “*soma*”, meaning body, as these patchy domains were initially thought to correspond to sites of endocytosis [12]. Subsequent studies failed to detect endocytosis at MCC/eisosomes [11,40,41,42,43]. However, eisosomes appear to play an indirect role in endocytosis because they influence lipid homeostasis and recruitment of proteins to the plasma membrane, as will be described further below in Section 4 [44]. The MCC and eisosome are connected parts of the same overall structure, as is diagrammed in Figure 2 along with representative proteins. At least 17 proteins appear to localize to eisosomes in *S. cerevisiae* (Table 1) [16,18,21,35,45,46].

A third line of work used electron microscopy to make the landmark discovery that MCC/eisosomes correspond to invaginations in the plasma membrane [14]. Analysis of freeze-etched *S. cerevisiae* plasma membranes showed that MCC/eisosomes correspond to furrows that are about 50 nm deep and 200 to 300 nm long. Furrows have been observed in the plasma membrane since the 1960s [47], but their significance was unclear other than that they were distinct from the finger-like projections that form during endocytosis [17,47,48,49,50,51]. Recent studies have shown that plasma membrane furrows can be found in a wide range of organisms with cell walls, including fungi, algae, and lichens [15]. However, their size and shape can be quite variable in these different organisms. For example, *Schizosaccharomyces pombe* furrows are about 1–2 µm in length [42].

### 2.2. Regulation of MCC/Eisosome Assembly and Disassembly

Studies in *S. cerevisiae* revealed that a pair of related proteins, Pil1 and Lsp1, promote the formation of MCC/eisosomes and the associated furrows [12,16,46,52]. Although *S. cerevisiae* Lsp1 is 70% identical to Pil1, it cannot function on its own as it does in some other species, most likely because it binds less efficiently to the plasma membrane [12,53]. Determination of the high-resolution structure of Lsp1 yielded important insights by showing that Pil1 and Lsp1 contain BAR domains that are known to bind and promote membrane curvature [53,54,55]. Another key discovery is that Pil1 and Lsp1 can assemble into long filaments. Taken together, this led to a “half-pipe” model for eisosome formation in which filaments of Pil1 and Lsp1 align their BAR domains to the plasma membrane, resulting in the formation of the furrow [39]. Although eisosomes are thought to be very stable, recent studies found that at least half of the eisosomes in the cell can undergo exchange of Pil1 subunits, most likely at the ends of the long filaments, indicating that some portions of eisosomes can undergo remodeling [56,57].

Additional proteins are thought to act with Pil1 to regulate MCC/eisosome formation and stability. For example, Seg1 promotes the formation of eisosomes by recruiting Pil1 to the plasma membrane [58]. This appears to be the limiting step in eisosome biogenesis. The MCC protein Nce102 and the eisosome proteins Slm1/2 are thought to influence eisosome formation by regulating the synthesis of lipids, especially sphingolipids and ergosterol [14,16,21,46].

Pil1 and Lsp1 are regulated in *S. cerevisiae* by a pair of redundant protein kinases, Pkh1 and Pkh2, that localize to eisosomes [30,31]. However, mutations affecting different groups of phosphorylation sites on Pil1 have yielded varying phenotypes, suggesting that regulation by phosphorylation may be complex [18,31,59,60]. A role for Pil1 phosphorylation in promoting eisosome disassembly was proposed based on an analysis of the effects of mutating four phosphorylation sites (Ser45 Ser59 Ser230 Thr233) that lie within the membrane-binding surface of Pil1. It seems likely that the negatively charged phosphate groups interfere with membrane association and prevent eisosome formation [21,31,54]. Interestingly, mutating a set of phosphorylation sites that primarily face away from the plasma membrane (Ser6 Thr27 Ser59 Thr233 Ser273 Ser299) led to the opposite conclusion that phosphorylation promotes eisosome assembly. These phosphorylation sites are therefore likely to be involved in promoting or stabilizing filament formation [54,59]. However, different interpretations have also been made concerning the effects of mutating just residues Ser230 and Thr233 [18,60], suggesting that growth conditions may also contribute to different phenotypes. In *C. albicans*, deleting the Pkh kinases promotes formation of long filaments of Pil1 and Lsp1, as well as long chains of membrane furrows, suggesting that phosphorylation promotes eisosome disassembly [19].

### 2.3. Spatial Regulation of MCC/Eisosomes

Eisosomes form somewhat randomly, however their spatial location is regulated. For example, MCC/eisosomes do not overlap, suggesting there is a mechanism that prevents the formation of a new MCC/eisosome too close to an existing one [34]. Also, new MCC/eisosome formation is restricted to zones of expanding morphogenesis, such as a growing bud, but not a mother cell [11,34]. The tip of new buds is initially devoid of MCC/eisosomes, as it takes time for these domains to assemble [9,34]. Another reason MCC/eisosomes do not overlap is that they do not diffuse in the plasma membrane [9,11,12]. The immobile nature of these domains does not appear to be due to a direct connection to the cell wall, actin filaments, or microtubules [10,42]. Perhaps the immobility of MCC/eisosomes is due to the formation of membrane furrows at these sites combined with the attached filaments comprised of thousands of copies of Pil1 and Lsp1. Other BAR domain-containing proteins have been shown to promote formation of stable lipid domains by “freezing” phosphoinositides [61], suggesting that Pil1 and Lsp1 could have a similar effect.

## 3. MCC/Eisosome Function in Cell Wall Synthesis and Morphogenesis

### 3.1. General Functions of MCC/Eisosomes

MCC/eisosomes play at least two general roles in the plasma membrane. One is to act as scaffolds that recruit specific proteins to these domains. Genome-wide GFP-tagging studies in *S. cerevisiae* have helped to identify proteins that localize to MCC/eisosomes (Table 1). Some of the proteins are very stably associated with the MCC/eisosome, such as the tetraspan protein Sur7 [11,62]. Other proteins can move in and out of these domains in response to signals. For example, Can1 is concentrated in MCC/eisosomes, but can diffuse out to regions where it can be endocytosed [16,29]. The Nce102 and Slm1/2 proteins can move out of these domains in response to sphingolipid levels [21]. The Xrn1 exonuclease resides in eisosomes until altered nutrient conditions permit its migration to the cytoplasm where it can promote the degradation of mRNAs at P bodies [36,63].

Another general function of MCC/eisosomes is to act as protected islands that stabilize proteins in the plasma membrane and protect them from endocytosis [16]. For example, the MCC protein Sur7 is one of the most stable proteins in *S. cerevisiae* cells [64]. Other MCC/eisosome functions are being investigated, such as the possible role of acting as a reservoir of membrane that could permit rapid expansion of the plasma membrane under stress conditions [23].

### 3.2. MCC/Eisosomes Regulate Spatial Organization of the Cell Wall and Morphogenesis

Mutation of MCC/eisosome proteins has been associated with altered cell wall synthesis in some species. For example, *S. cerevisiae pil1*∆ mutants and *C. albicans pil1*∆ *lsp1*∆ mutants produce broad invaginations of cell wall material [12,19]. Interestingly, the effect is more extreme in *C. albicans*; both *pil1*∆ *lsp1*∆ and *sur7*∆ mutants form deep invaginations of cell wall [19,62]. In *C. albicans*, many of the invaginations are in the form of long tubes of cell wall material [62]. Interestingly, similar tubular cell wall invaginations were observed in a *C. albicans inp51*∆ mutant, which lacks a PI_5_-specific PI_4,5_P_2_ phosphatase [65], suggesting that the cell wall invaginations are linked to abnormal regulation of PI_4,5_P_2_. Studies in *S. cerevisiae* and *S. pombe* have also linked MCC/eisosomes to the regulation of PI_4,5_P_2_ [20,22,66], raising the possibility that this is a common function of MCC/eisosomes. The abnormal cell wall phenotype of the *C. albicans pil1*∆ *lsp1*∆ mutant could be partially rescued by overproducing Sur7, which suggested that Sur7 plays the key role in regulating PI_4,5_P_2_ levels, and that the main role for eisosomes in regulation of the cell wall is to stabilize Sur7 at the plasma membrane [19].

In addition to abnormal cell wall localization, the *C. albicans sur7*∆ and *pil1*∆ *lsp1*∆ mutants showed abnormal morphogenesis, as they displayed defects in undergoing the highly polarized morphogenesis required to form hyphae [19,62]. Abnormal hyphal morphogenesis was also seen in an *Ashbya gossypii pil1*∆ mutant [41]. The *C. albicans sur7*∆ and *pil1*∆ *lsp1*∆ mutants were also interesting in that they formed extremely large mother cells, which was likely due in part to disruption of actin filaments and localization of the cortical actin patches to the mother cells instead of to the growing buds, as expected. In addition, septin proteins were found to be present at other sites in the plasma membrane, often forming small ectopic rings, rather than being restricted to the bud neck as they would be in wild type cells [67]. Abnormal septin localization could contribute to the altered cell wall synthesis, as septins are well known to recruit cell wall synthesis machinery when they are in their typical location at the bud neck [68]. The role of septins will be described further in Section 4.1.

### 3.3. C. albicans sur7*∆* Mutant Makes Thicker, but Weaker Cell Walls

**Figure 3:**
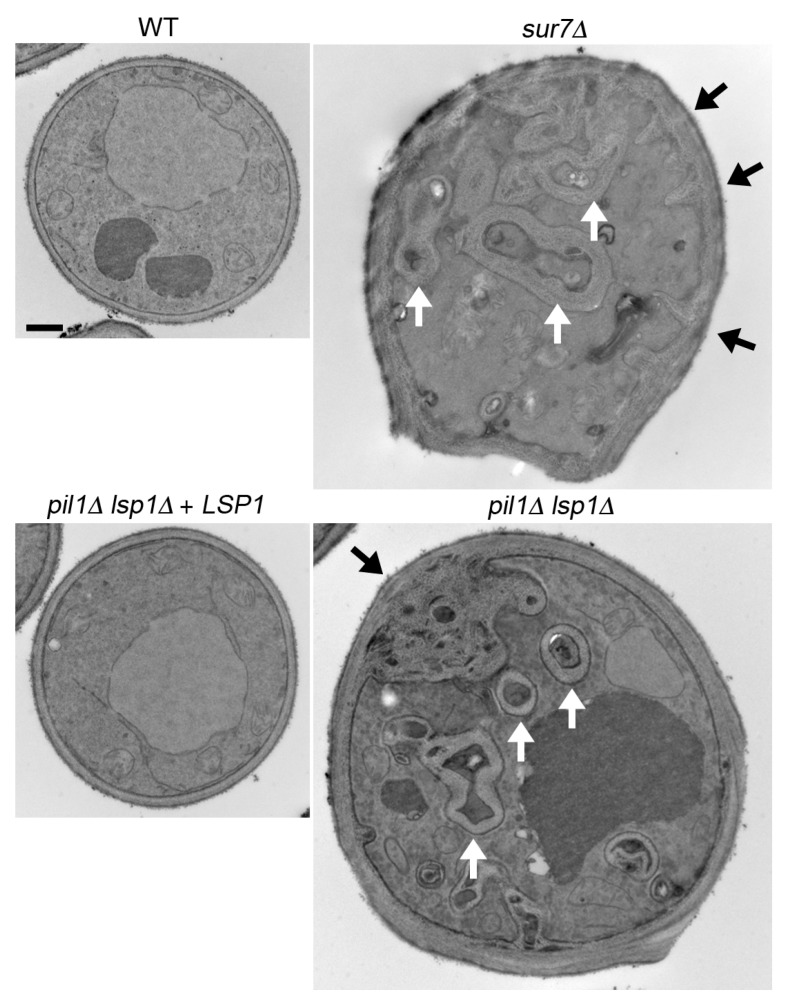
Abnormal cell wall invaginations in *C. albicans sur7*∆ and *pil1*∆ *lsp1*∆ mutants. The indicated cell sections were analyzed by transmission electron microscopy. Thicker cell walls and invaginations of cell wall material were detected in both the *sur7*∆ mutant and the *pil1*∆ *lsp1*∆ double mutant. The white arrows indicate tubes of cell wall material. Black arrows indicate spots where there are spiky invaginations in the *sur7*∆ mutant and the large round cell wall invagination in the *pil1*∆ *lsp1*∆ mutant. Black bar, 1 µm. This image was reproduced from Figure 2 of the paper by Wang et al. [19].

EM studies demonstrated that the *C. albicans pil1*∆ *lsp1*∆ and the *sur7*Δ mutants formed thicker cell walls (Figure 3) [19,62,69]. In spite of this, the mutant cell walls were defective as the cells showed increased sensitivity to factors that exacerbate cell wall defects [19,62,69,70]. The *sur7*∆ mutant cell walls may be weaker because they were shown to contain lower levels of β-glucan, which is important for cell wall rigidity [70]. Sur7 presumably influences β-glucan synthesis indirectly, as it has been shown that the β-1,3-glucan synthase enzyme is mobile and often associated with cortical actin patches rather than the static MCC/eisosomes [71,72,73]. Thicker, but weaker, cell walls were also reported for mutant cells of *Beauveria bassiana*, an insect fungal pathogen, lacking PilA or PilB [74].

### 3.4. Abnormal Regulation of PI_4,5_P_2_ Contributes to the Altered Cell Wall Phenotype of Eisosome Mutants

MCC/eisosomes have been implicated in regulating the homeostasis of different kinds of lipids including PI_4,5_P_2_ [19,20,22] and sphingolipids [21,75,76]. PI_4,5_P_2_ is significant because it is a key regulatory lipid that can influence cell wall synthesis and morphogenesis. *S. cerevisiae* eisosomes regulate PI_4,5_P_2_ by recruiting the lipid phosphatases Inp51 and Inp52 to the plasma membrane, which then decrease PI_4,5_P_2_ by dephosphorylating it and converting it to PI_4_P [20,44]. The *S. pombe* eisosomes have also been linked to regulation of PI_4,5_P_2_ by genetic studies indicating that Pil1 acts in a genetic pathway with Syj1, a synaptojanin-like lipid phosphatase that can dephosphorylate PI_4,5_P_2_ similar to Inp51 and Inp52 in *S. cerevisiae* [22,66].

Studies with *C. albicans* have linked the MCC protein Sur7 to the regulation of PI_4,5_P_2_ levels. Both a *sur7*∆ mutant and a *pil1*∆ *lsp1*∆ mutant displayed elevated levels of PI_4,5_P_2_ in the plasma membrane at sites of abnormal cell wall invaginations [19]. A similar phenotype was reported for a *C. albicans* mutant lacking the Inp51 PI_4,5_P_2_ phosphatase [65], further supporting the conclusion that elevated PI_4,5_P_2_ levels promotes abnormal cell wall synthesis. The sites of elevated PI_4,5_P_2_ are thought to act by promoting abnormal recruitment of proteins that control cell wall synthesis, such as the septin proteins. Interestingly, overexpression of *SUR7* strongly rescued the abnormal PI_4,5_P_2_ and cell wall properties of *pil1*∆ *lsp1*∆ mutants [19]. This indicates that *C. albicans* eisosomes function to promote the stability of Sur7 at the plasma membrane so that it can properly regulate PI_4,5_P_2_ [19].

### 3.5. MCC/Eisosomes Contribute to Invasive Growth and Virulence of C. albicans

Proper cell wall synthesis and morphogenesis promoted by MCC/eisosomes is important for *C. albicans* virulence, as the *sur7*∆ mutant showed a greatly reduced virulence in a mouse model of systemic infection [77]. One contributing factor is that *sur7*∆ cells were defective in forming hyphal filaments that promote invasive growth into tissues in vivo and for forming biofilms [62,69,70,77]. It is also likely that the altered cell wall produced by *sur7*∆ mutants resulted in unmasking of β-glucans and other cell wall structures that are recognized by the innate immune system. Interestingly, Sur7-GFP was detected at sites where neutrophils attacked *C. albicans*, suggesting that, in wild type cells, Sur7 is involved in producing a distinct type of cell wall material at these sites that is more readily recognized by the immune system [78]. Another contributing factor to the virulence defect of *sur7*∆ mutant cells is that they are more sensitive to a variety of stresses encountered in vivo, as will be described below in Section 5.

Another MCC domain protein important for *C. albicans* virulence is Nce102 [79]. A distinctive property of *nce102*∆ mutant cells is that they fail to grow invasively into low concentrations of agar that are soft, but invade well into high concentrations of agar that are more rigid. This was unexpected, since mutants with defects in invasive growth are expected to have greater difficulty invading with increased density of the agar matrix [80]. This suggests that a denser agar matrix provided a second signal to stimulate the *nce102*∆ mutant cells to undergo invasive hyphal growth. This unique invasive growth defect of *nce102*∆ cells appears to be due to a partial defect in actin organization [79]. These virulence defects highlight the potential significance of MCC/eisosomes as novel drug targets.

## 4. MCC/Eisosomes Affect Other Plasma Membrane Domains That Can Influence Cell Wall Synthesis

### 4.1. Septins Regulate Cell Wall Synthesis during Cytokinesis and Polarized Morphogenesis

MCC/eisosomes are important for the proper organization of other regions of the plasma membrane that are outside of these patchy domains. For example, septin proteins mislocalize to clusters found throughout the plasma membrane in *C. albicans sur7*∆ and *pil1*∆ *lsp1*∆ mutants [19,62]. Septins were discovered in *S. cerevisiae* for their role in promoting septation [68,81]. These GTP-binding proteins assemble into filaments that form a ring on the inner surface of the plasma membrane prior to bud emergence. The bud forms through the septin ring, and then the septin ring acts as a scaffold to recruit proteins that synthesize the cell wall material to form the septum. Thus, altered septin localization is thought to contribute to the cell wall and morphogenesis phenotypes of MCC/eisosome mutants. In fact, the septins often form small rings in the plasma membrane that may be responsible for nucleating the tubes of cell wall invaginations seen in *C. albicans sur7*∆ and *pil1*∆ *lsp1*∆ mutants (Figure 3 and Section 3.2) [19,62]. This possibility is supported by the fact that some *S. cerevisiae* septin mutants form abnormal cell wall invaginations [82,83].

Studies in *S. cerevisiae* have shown that septins function in at least two ways: as a scaffold to recruit proteins and as a barrier in the plasma membrane to prevent diffusion [84]. Many of the ~60 proteins that localize to the bud neck in a septin-dependent manner are involved in processes other than septation, such as the Bud proteins that act to select the future site of bud emergence, Bni4 that recruits chitin synthase to form a chitin ring at the future bud site, Swe1 that acts in a cell cycle checkpoint, and proteins involved in sensing spindle orientation [85,86,87]. The septin barrier function is important to prevent membrane proteins and cortical ER in the bud from diffusing back into the mother cell, which maintains polarized growth in the bud [88,89,90,91]. This barrier function also helps to restrict septum formation to the neck region [68,85].

In addition to acting in septation, septins guide proper cell wall synthesis during different types of highly polarized cell growth [92]. For example, septins act in pheromone-induced morphogenesis in *S. cerevisiae* to promote formation of the conjugation bridge that connects mating cells [93,94]. In *C. albicans*, *cdc10*∆ and *cdc11*∆ septin mutants develop abnormally curved hyphal filaments, indicating a role in guiding polarized tip growth that is perhaps mediated by a patch of septins at the leading edge of hyphal growth [80,95,96]. The *cdc10*∆, *cdc11*∆, and a *cdc12-6^ts^* mutants also have defects in selecting sites for new hyphal outgrowths, which may limit the ability to disseminate infection [80,97,98]. In the rice blast fungus *Magnaportha oryzae*, septins are important for the invasive appressorium [99]. Septins have also been implicated in other types of specialized morphogenesis in a variety of different plant and animal pathogens [67,92,100,101].

### 4.2. Sites of Secretion

The docking of secretory vesicles with special domains in the plasma membrane is a complex process that must be coordinated efficiently for proper cell wall synthesis [102,103,104]. Secretory vesicles emanating from the Golgi are guided along actin cables by the myosin-V motor protein Myo2p to target sites in the plasma membrane. The exocyst complex of proteins tethers the vesicle to the membrane, and then the SNARE proteins mediate fusion [102,103,105,106]. The exocyst proteins, Sec3 and Exo70, arrive at the plasma membrane first and then direct the future docking of secretory vesicles. Both of these proteins associate with PI_4,5_P_2_ in the plasma membrane [107]. Small GTPases are also needed to recruit Sec3 and Exo70 to the target site [102,108,109]. The abnormal cell wall and morphogenesis defects seen in MCC/eisosome mutants are therefore likely due in part to less efficient targeting of secretory vesicles to sites of polarized growth, caused by abnormal actin localization and PI_4,5_P_2_ levels.

### 4.3. Sites of Endocytosis in the Plasma Membrane

Endocytosis is important to bring nutrients and other substances into the cell and for the turnover and homeostasis of lipids and proteins in the plasma membrane [110,111,112]. The most well understood endocytic pathway is mediated by clathrin. In *S. cerevisiae*, this complex pathway involves the ordered recruitment of >50 proteins. Current models divide the endocytic process into several modules. (i) The Early module begins as a patch of proteins in the plasma membrane that develops into the (ii) Early Coat module when clathrin forms a lattice on the inner surface of the plasma membrane and begins the process of invaginating the membrane to develop a vesicle [113]. As additional proteins are recruited, this domain transitions through the (iii) Intermediate Coat stage, the (iv) Late Coat stage, and then the (v) WASP/MYO stage. This module forms with the recruitment of Las17, the WASP protein in yeast, which serves as the chief inducer of the Arp2/3 complex nucleation of actin filament polymerization [114]. The next major event is the arrival of (vi) actin and its accompanying factors to form the Actin module. Induction of the Arp2/3 complex by Las17p begins actin nucleation and polymerization, providing the driving force for membrane invagination [110,111]. The scission phase then releases the endocytic vesicle.

Cortical patches of actin associated with the inner surface of the plasma membrane, one of the hallmarks of sites of endocytosis, are distinct from MCC/eisosomes. As previously mentioned, MCC/eisosomes were initially thought to correspond to sites for an alternative type of endocytic pathway [12], but other studies failed to find any overlap with sites of endocytosis [11,12,40,41,42,43]. However, there are several interesting connections between endocytosis and MCC/eisosomes. One is that *S. cerevisiae pil1*∆ mutants display a defect in endocytosis, which appears to be due to the failure to recruit some components of the endocytic machinery to the plasma membrane [44]. Also, *SUR7* was identified because its overexpression suppressed the growth defects of an *S. cerevisiae rvs167* mutant, which is defective in the scission phase of endocytosis [115]. *C. albicans sur7*∆ mutants undergo efficient internalization of vesicles, but show a late defect in trafficking vesicles to the vacuole, presumably due to altered actin filaments [62]. Thus, defects in endocytosis may contribute to the altered spatial regulation of cell wall synthesis and endocytosis defects of MCC/eisosome mutants.

### 4.4. Sites of Contact between the Endoplasmic Reticulum (ER) and Plasma Membrane

Sites of direct contact between the cortical endoplasmic reticulum (ER) and plasma membrane form a specialized domain that is important for proper cellular organization and function [8,90,116]. Although the ER is best known for its role in the early stages of the secretory pathway, the cortical ER also forms direct contact with the plasma membrane and other membrane bound organelles [116,117]. It is estimated that around 65% of the *S. cerevisiae* plasma membrane is covered by ER [118]. The cortical ER is very close to the plasma membrane at these sites, as they are only about 33 nm apart [119,120]. Thus, the ER acts as a barrier that prevents regions of the plasma membrane from associating with cytoplasmic proteins, thereby blocking secretion and endocytosis at these sites [118]. Interestingly, the ER does not form contacts with the plasma membrane at MCC/eisosome domains [118], indicating that mutants lacking MCC/eisosomes will be able to form ER-plasma membrane contact sites over a broader range of the cell cortex.

The contact sites between the cortical ER and the plasma membrane have key roles in lipid synthesis and the homeostasis of ions and lipids [117]. This close contact allows the transfer of lipids, proteins, and signals to flow directly from the ER to the plasma membrane. For example, sphingolipids and phospholipids synthesized at these sites are directly transferred to the plasma membrane, bypassing the Golgi and secretory vesicles [117,120,121]. Another example is that the Sac1 lipid phosphatase is anchored in the ER, but acts to dephosphorylate PI_4_P in the plasma membrane, preventing it from being converted into the regulatory lipid PI_4,5_P_2_ [122]. Sac1 also functions in lipid homeostasis by acting in concert with Osh6 to allow phosphatidylserine to traffic from its site of synthesis in the ER to the plasma membrane [123,124].

## 5. MCC/Eisosomes Protect Against Stress

### 5.1. Cell Wall Stress

The phenotypes of MCC/eisosome mutants indicate that they play a role in mitigating cell wall stress. As mentioned previously, *C. albicans sur7*Δ cells have increased sensitivity to cell wall perturbing conditions like treatment with Calcofluor White or elevated temperature [62,69,70]. Interestingly, *C. albicans* Sur7 has been shown to be recruited to sites of neutrophil attack, suggesting a role for MCC/eisosomes in the new cell wall synthesis that occurs in response to this type of cell wall damage [78]. Although in *S. cerevisiae* a *sur7*∆ mutation causes weaker phenotypes than in *C. albicans*, related observations have been reported, such as greater susceptibility to osmotic stress [125]. Other work with *S. cerevisiae* demonstrated that Pun1, a paralog of Sur7, is induced upon cell wall damage, and *pun1*∆ mutant cells have modified cell walls [126]. The *pun1*∆ cells are more sensitive to the cell wall degrading enzyme zymolyase, which correlates with other results indicating that the mutant cells have reduced levels of 1,3-β-d-glucans and mannoproteins [126]. Eisosomes have also been shown to play a role in surviving hypo-osmotic stress in *S. pombe* protoplasts [23]. Taken together, these results indicate that MCC/eisosomes are important for maintaining cell wall strength, although future studies will be needed to determine if this role is direct or indirect since sites of cell wall synthesis do not appear to overlap with eisosomes.

### 5.2. Lipid Homeostasis-Related Stress

An imbalance in the proper composition and distribution of lipids in the plasma membrane can result in disrupted signal transduction, endocytosis, and transport [127,128,129]. As mentioned above, MCC/eisosomes have been implicated in regulating the homeostasis of different lipids. Nce102 is enriched in *S. cerevisiae* MCC/eisosomes and acts as a component of sphingolipid sensor machinery [21,130]. Nce102 moves in and out of the MCC/eisosome in response to sphingolipid levels in order to interact with Sng1 and thus regulate the Pkh-Ypk signaling module [21,130]. This regulation results in the generation of more sphingolipids. Additionally, a lack of sphingolipids causes Slm1/2 to leave eisosomes and associate with TORC2 [75,76]. Slm1/2 then recruits YPK1, which phosphorylates the Orm1/2 proteins and allows sphingolipid synthesis to occur [131,132,133,134,135]. As discussed above, the *S. cerevisiae* lipid phosphatases Inp51 and Inp52 are recruited by MCC/eisosomes to dephosphorylate PI_4,5_P_2_ and convert it to PI_4_P [20], which reduces the phosphorylation of lipids.

### 5.3. Copper/Metal Ion-Induced Stress

MCC/eisosomes contain proteins that promote resistance to stress caused by excessive amounts of toxic metal ions. For example, *C. albicans sur7*Δ cells are about 1000-fold more sensitive to copper [77]. This increased susceptibility to copper correlated with poor growth in macrophage phagosomes. Recent studies have shown that stimulated macrophages translocate the ATP7A copper transporter to the phagosomal membrane so that it pumps copper into the phagosome as part of the antimicrobial response [136]. Consistent with this, other copper sensitive mutants of *C. albicans* also showed defects in phagosomal growth [77]. In *S. cerevisiae*, *PUN1* expression is induced by heavy metal ion stress through the Crz1 transcription factor. Deletion of *PUN1* results in altered sensitivity to varying metal ions including manganese, arsenic, nickel, and calcium [126]. These *pun1*Δ mutants also have an altered morphology and cell wall composition (see Section 5.1).

### 5.4. Oxidative Stress

Oxidative stress is a common insult faced by microbial pathogens from the host immune system, typified by the neutrophil’s oxidative burst. A family of four eisosome proteins (Pst1, Pst2, Pst3, and Ycp4) in *C. albicans* was recently shown to carry out a novel antioxidant function [137]. They are related to flavodoxin-like proteins (FLPs), which act as NAD(P)H:quinone oxidoreductases. It was proposed that in *C. albicans* these eisosome proteins function by reducing ubiquinone so that it can act as an antioxidant to prevent damage to the plasma membrane [137]. An advantage is that ubiquinone can prevent oxidative damage within the plasma membrane, such as lipid peroxidation, which could not be readily accessed by previously identified antioxidant enzymes, including catalase and superoxide dismutase [138,139,140,141]. Consistent with an important role for these eisosome proteins, the quadruple mutant (*pst1*Δ, *pst2*Δ, *pst3*Δ, *ycp4*Δ) was avirulent in a mouse model of disseminated *C. albicans* infection [137]. Similar FLPs (Rfs1, Pst2, Ycp4) are present in *S. cerevisiae*, and there is some evidence that they can promote resistance to oxidative stress [142,143,144]. In vitro analysis of Pst2 showed that it can act as an NAD(P)H:quinone oxidoreductase to reduce a variety of quinones, including 1,4-benzoquinone [145].

## 6. Concluding Comments

MCC/eisosome furrows represent a novel type of domain that has broad roles in regulating plasma membrane function. Interestingly, furrows were detected in the plasma membrane of a wide range of fungi and microalgae that have cell walls, but not in closely related organisms that do not [15]. This suggests MCC/eisosome function is particularly important for walled organisms. Consistent with this, mutants of several species of fungi that lack MCC/eisosomes displayed defects in cell wall synthesis and morphogenesis. However, the role of specific MCC/eisosome proteins, such as Sur7, is variable in different organisms. This suggests that in some organisms MCC/eisosome proteins may have become specialized in different ways, while preserving the overall function of these domains. A key goal for future studies will therefore be to investigate MCC/eisosomes in a broader range of fungi and algae. Comparative studies will help to identify novel mechanisms that regulate cell wall synthesis and plasma membrane function.

## Figures and Tables

**Table 1:** *S. cerevisiae* MCC/eisosome proteins.

Protein	Location	Function	Localization Reference	Copies/cell ^1^
Sur7	MCC	Sur7 family tetraspan	[9,11]	17,000
Fmp45	MCC	Sur7 family tetraspan	[11]	329
Pun1	MCC	Sur7 family tetraspan	[16]	1660
Ynl194c	MCC	Sur7 family tetraspan	[11]	ND
Nce102	MCC	Nce102 family tetraspan	[16]	1824
Fhn1	MCC	Nce102 family tetraspan	[16]	ND
Can1	MCC	H^+^-driven Arg permease	[9]	ND
Fur4	MCC	H^+^-driven uracil permease	[10]	3
Tat2	MCC	H^+^-driven Trp and Tyr permease	[28]	752
Pil1	eisosome	BAR domain	[12]	115,000
Lsp1	eisosome	BAR domain	[12]	104,000
Pkh1	eisosome	Ser/Thr protein kinase	[30,31]	221
Pkh2	eisosome	Ser/Thr protein kinase	[30,31]	229
Eis1	eisosome	Unknown	[16]	5570
Slm1	eisosome	BAR domain and PH domain	[16,32]	5190
Slm2	eisosome	BAR domain and PH domain	[16,32]	2610
Seg1	eisosome	Unknown	[18,33]	982
Mdg1	eisosome	Unknown	[16]	1240
Ygr130c	eisosome	Unknown	[16,18]	10,300
Pst2	eisosome	Similar to flavodoxin-like proteins	[16]	2330
Rfs1	eisosome	Similar to flavodoxin-like proteins	[16]	7060
Ycp4	eisosome	Similar to flavodoxin-like proteins	[16]	14,600
Msc3	eisosome	Protein of unknown function	[34,35]	131
Xrn1	eisosome	Exonuclease	[36]	11,700

^1^ Copies of proteins per cell from data reported by [37,38]. ND indicates no data available.
